# Adenoid cystic carcinoma of maxillary sinus: report of two cases

**DOI:** 10.11604/pamj.2024.48.113.44090

**Published:** 2024-07-17

**Authors:** Anouar Ben Ameur El Youbi, Mohamed Bouqes, Najib Benmansour, Mohammed Ridal, Nawal Hammas, Mohamed Noureddine EL Alami El Amine

**Affiliations:** 1 Department of Otolaryngology and Cervicofacial Surgery, Hassan II University Hospital Center, Fes, Morocco,; 2 Anatomopathology Laboratory, Hassan II University Hospital Center, Fes, Morocco

**Keywords:** Adenoid cystic carcinoma, paranasal sinuses, palate, case report

## Abstract

Adenoid cystic carcinoma infrequently affects paranasal sinuses. It's a slowly progressing, aggressive cancer with a tendency to invade nerves. The research underscores the significance of prompt diagnosis and effective management of adenoid cystic carcinoma. Two cases of adenoid cystic carcinoma of the maxillary sinus are presented. The first, a 73-year-old woman, presented with right nasal obstruction. The second, a 53-year-old woman, presented with a hard palatal mass and right nasal obstruction. The biopsy confirmed adenoid cystic carcinoma in the first patient. Given the extent of the tumor, she was referred to radiotherapy to complement the therapeutic treatment. The second patient underwent mass excision, also confirmed to be adenoid cystic carcinoma, followed by radiation therapy. Adenoid cystic carcinoma of the maxillary sinus is often diagnosed at an advanced stage due to its slow growth and local regional spread, making its diagnosis and therapeutic management particularly challenging.

## Introduction

Adenoid cystic carcinomas are malignant tumors, accounting for 5% of all malignant cancers of the paranasal sinuses, which represent less than 0.15% of all malignant head and neck cancers, regardless of site and histology [1]. This tumor has a tendency to progress insidiously, often being diagnosed at an advanced stage, which is correlated with an unfavorable prognosis for the patient [2]. Histopathological analysis is essential for establishing a precise diagnosis [3]. Surgical intervention with curative intent followed by adjuvant radiotherapy as the primary approach for treating adenoid cystic carcinoma [4]. We present two cases of adenoid cystic carcinoma of the maxillary sinus. The first case exhibited extensive tumor extension, leading to referral for radiotherapy alone as an adjunct to management. The second case underwent surgical excision of the tumor followed by radiotherapy.

## Patient and observation

**Patient information:** a female patient, 73-years-old of age, with no personal medical history or family history of carcinoma, presenting with facial asymmetry, notably a slight swelling in the right cheek region associated with partial obstruction of the ipsilateral nasal fossa evolving over four years.

**Figure 1:**
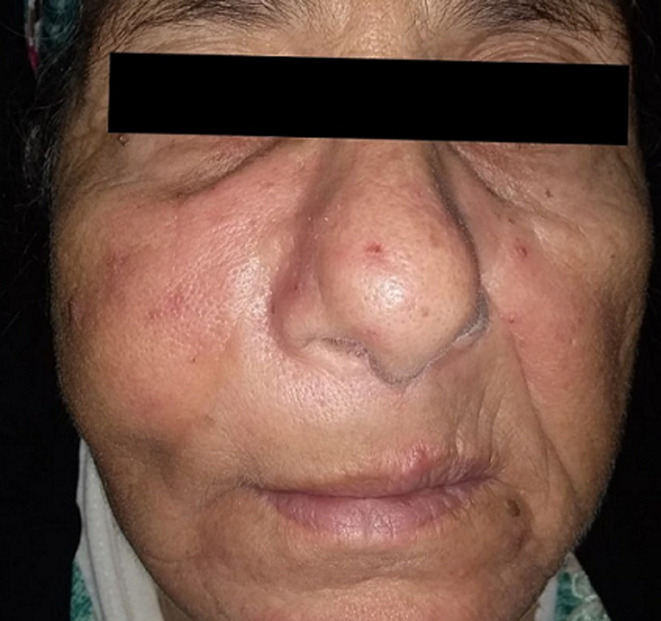
clinical image showing a slight swelling on the right cheek

**Clinical finding:** the clinical examination reveals a slight swelling in the right maxillary region, without inflammatory signs, painless on palpation, associated with hypoesthesia of the ipsilateral V2 nerve (maxillary nerve) (Figure 1). Rhinological examination shows swelling of the medial wall of the nasal cavity with partial nasal obstruction on the right side. The rest of the clinical examination is unremarkable.

**Figure 2:**
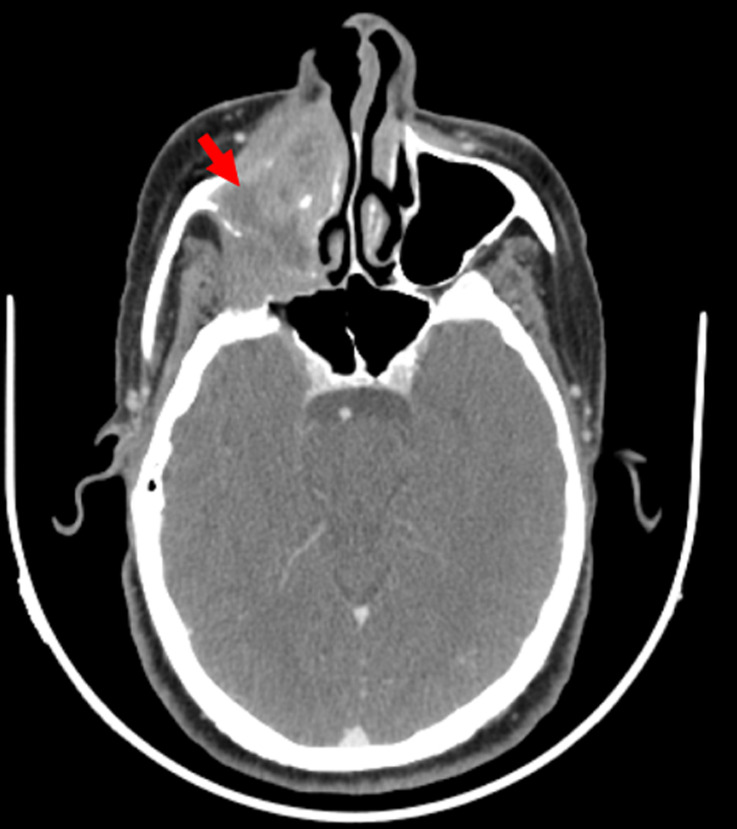
axial computerized tomography scan image shows an advanced tissue process localized within the right maxillary sinus (red arrow)

**Diagnostic and assessment:** craniofacial computed tomography showed the presence of a tissue process centered on the right maxillary sinus, measuring 58 cm in its greatest axis. Topographically, this process erodes the anterior wall of the maxillary sinus, as well as the nasal root, apex of the maxillary sinus, and pterygoid process with invasion of the ipsilateral medial and lateral pterygoid muscles. It also causes focal erosion of the anterior wall of the right sphenoidal sinus without clear signs of endosinusal invasion. Additionally, it erodes the floor of the right orbit with extension and invasion of the extraconal fat and ipsilateral inferior rectus muscle. There is also an extension into the orbit via the inferior orbital fissure with close contact with the optic nerve at this level (Figure 2).

**Figure 3:**
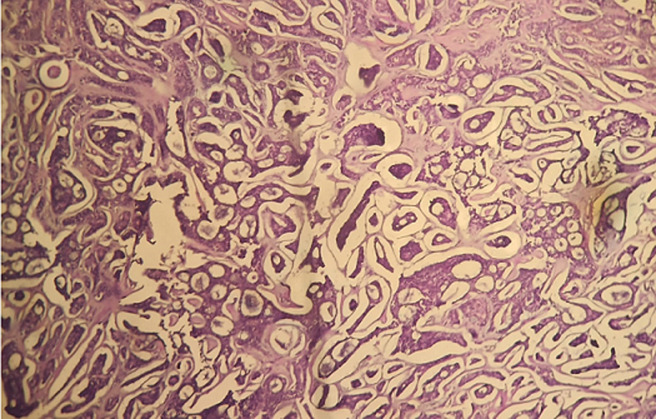
image of adenoid cystic carcinoma displaying a tumor proliferation characterized by highly fibrous stroma and perineural sheathings, magnified at 100x

**Therapeutic intervention:** biopsy of the mass performed via the vestibular approach of Caldwell-Luc returned a pathological diagnosis in favor of a maxillary sinus adenoid cystic carcinoma. Histologically, there is a tumor proliferation arranged in tubules, trabeculae, and cribriform masses. The tumor cells have a basaloid appearance, with round or ovoid nuclei, fine chromatin, and scanty amphophilic cytoplasm. The stroma is highly fibrous. Perineural sheathings are present (Figure 3). Due to extensive local infiltration, the patient was referred for radiotherapy to complement her therapeutic management.

**Follow-up and outcomes:** the patient received radiotherapy to the palate using the simultaneous integrated boost (SIB) technique with intensity-modulated radiation therapy (IMRT). She received 70 Gray in 35 fractions with a radiation course duration of 7 weeks.

**Patient perspective:** during the therapeutic management, the patient was satisfied with the level of care provided to her.

**Informed consent:** the patient gives his consent for the publication.

### Case report 2

**Patient information:** a 53-year-old female patient with no personal medical history or family history of carcinoma, who sought medical attention for the management of a swelling on the hard palate, evolving over a three-year period.

**Clinical finding:** clinical examination revealed a swelling on the hard palate, lateralized to the right, painless, and fixed, without inflammatory signs. Rhinological examination revealed a bulging of the medial wall of the nasal fossa with obstruction in the right nasal fossa. The remainder of the clinical examination was normal.

**Figure 4:**
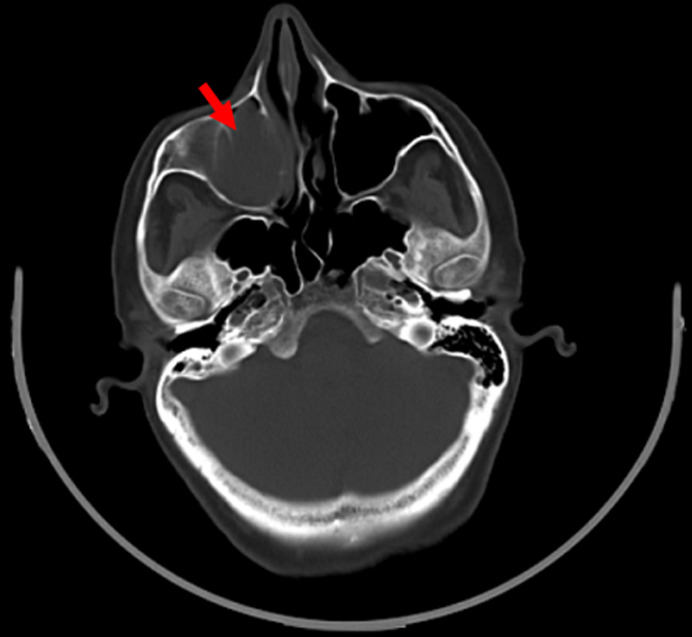
axial computerized tomography scan image depicting a tissue process centered within the right maxillary sinus (red arrow)

**Figure 5:**
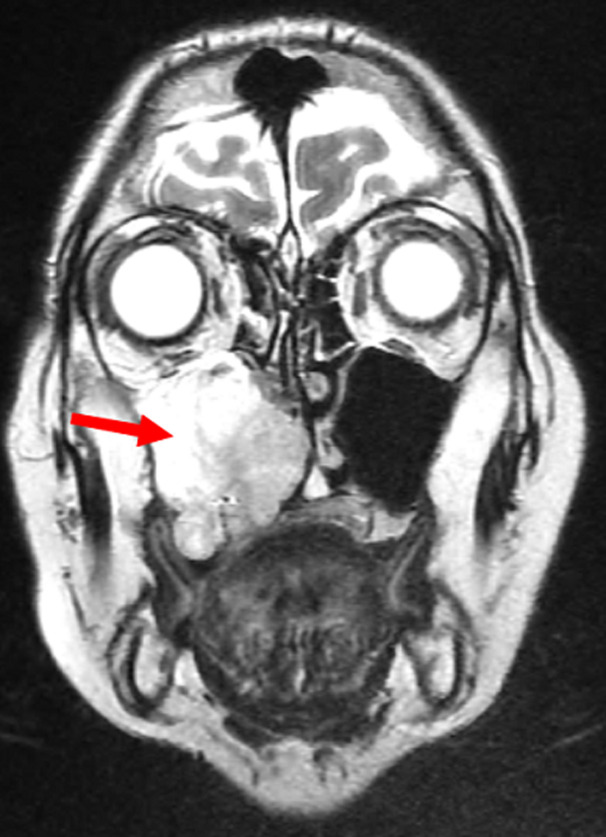
coronal section of the magnetic resonance imaging showing a tissue lesion in the right maxillary sinus (red arrow) extending into the endobuccal region and the ipsilateral nasal cavity

**Diagnostic and assessment:** a computed tomography scan detected a hypodense lesion in the right maxillary sinus, which exhibited intense enhancement after contrast administration, measuring 03x02 cm in diameter. This lesion extended into the right hard palate, contacting the nasal septum, which showed erosion inferiorly and laterally. It extended to the posterior portion of the right upper dental arch with associated bone lysis. Additionally, there were no observed cervical lymphadenopathies (Figure 4). Subsequently, the patient underwent magnetic resonance imaging, which showed a lesion centered on the right maxillary sinus, heterogeneously enhancing after Gadolinium, measuring 45x45x47 mm. It extended into the ipsilateral nasal cavity, displacing the middle and inferior turbinates, eroding the floor of the maxillary sinus inferiorly, and showing endobuccal extension (Figure 5).

**Figure 6:**
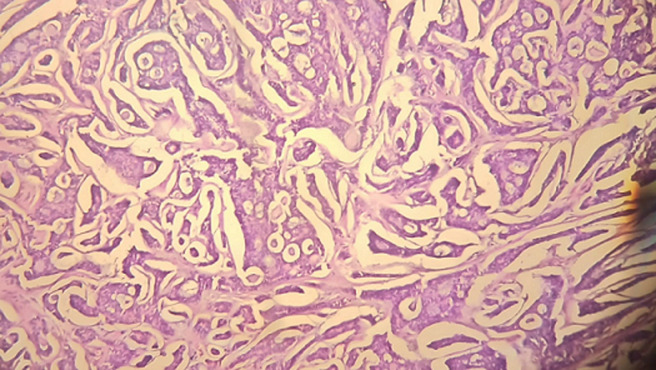
histological image of adenoid cystic carcinoma reveals a tumor proliferation characterized by cribriform masses, magnified at 100x

**Therapeutic intervention:** complete excision of the maxillary mass was performed via a para-lateral-nasal approach. Postoperative recovery was uneventful. Histologically, it is a nasal-sinus mucosa with a tumor proliferation arranged in sheets, trabeculae, tubes, and cribriform masses, sometimes cystic with round lumens containing pale eosinophilic material. The tumor cells are monomorphic, rounded, and small in size, with round or angular nuclei, homogeneous chromatin, and scanty eosinophilic cytoplasm. Some cells resemble myoepithelial cells. Mitoses are rare. The tumor stroma is scanty. No perineural sheathings are observed. An immunohistochemical study was performed. The aforementioned tumor cells express cluster of differentiation 117 (CD117), amyloid-like material (AML) particularly in diffuse areas and the peripheral pericanalicular zone, and focal epithelial membrane antigen (EMA). In conclusion, the histological and immunohistochemical features are compatible with a salivary gland-type adenoid cystic carcinoma (Figure 6). The patient was referred for radiotherapy to complete the therapeutic treatment, receiving a dose of 70 Gray in the right maxillary sinus and 56 Gray in the right cervical lymph nodes (zones Ib, II, III, and IV).

**Follow-up and outcomes:** follow-up computed tomography showed regression of the lytic lesion process in the right palate and maxillary sinus, with the presence of hypodense infiltration in the corresponding area. Clinical progress has shown the absence of tumor recurrence, and an improvement in symptoms over a four-year follow-up period.

**Patient perspective:** “I was handicapped and anxious because of the nasal obstruction and palatal mass. After surgical excision and radiotherapy, I am very satisfied with the improvement in clinical symptoms and the absence of recurrence.”

**Informed consent:** the patient gives his consent for the publication.

## Discussion

Adenoid cystic carcinoma originates from secretory glands [5]. It is a slow-growing malignant tumor characterized by extensive local infiltration, perineural spread, a propensity for local recurrence, and distant metastases [6]. Its occurrence in the maxillary sinus is rare, comprising only 0.3% to 1.0% of all sinus and nasal cavity cancers [5]. It is presumed to originate from the small seromucous glands within the mucosa, beneath the respiratory epithelium of the nasal cavity and paranasal sinuses [4]. Women are slightly more affected than men (with a female-to-male ratio of approximately 3:2), particularly between the ages of 30 and 70, and no proven risk factors have been identified [7]. Symptoms can vary, including nasal obstruction, epistaxis, and chronic pain may also occur [8]. Furthermore, depending on the growth and extent of the tumor, displacement of adjacent structures with gradual and painless swelling of the cheek region may be observed [8]. Moreover, this tumor can progress insidiously, often diagnosed at an advanced stage, which is associated with an unfavorable prognosis for the patient [2]. The involvement of swellings, particularly in the palate region, is also described in the literature [2].

Various diagnostic methods have been described for this tumor, such as computed tomography (CT) and magnetic resonance imaging (MRI), allowing visualization of the tumor's local expansive and destructive characteristics, as well as potential distant metastases [9]. Histopathological examination is necessary to establish an accurate diagnosis [3]. Histopathologically, the tumor exhibits invasive characteristics involving bone, neural structures, and lymphovascular channels, presenting diverse growth patterns such as cribriform, tubular, and solid formations. The cribriform subtype is associated with a more favorable prognosis compared to the solid subtype [9]. In the differential diagnosis of Adenoid cystic carcinoma, one may consider low-grade polymorphous adenocarcinoma, basaloid squamous cell carcinoma, small cell neuroendocrine carcinoma, and adenosquamous carcinoma [9]. Treatment choice depends on the site, stage, grade, and behavior of the tumor [4]. Most authors consider curative-intent surgery and adjuvant radiotherapy as the cornerstone of adenoid cystic carcinoma treatment. Surgery is deemed most effective when a clear margin of at least 2 mm around the tumor is achieved. Postoperative radiotherapy appears to have a significant impact on recurrence rates [4]. Chemotherapy has limited efficacy in this disease because Adenoid cystic carcinoma cells have low mitotic indices [10].

## Conclusion

Adenoid cystic carcinoma involving the maxillary sinus is a rare malignant tumor often detected at advanced stages due to its slow and regional spread, as observed in the first patient. This poses significant challenges for both diagnosis and treatment interventions. So, it should be considered in the differential diagnosis for patients presenting with nasal obstruction and slowly increasing swelling in and around the nasal cavity.
